# Antioxidant and Anti-Inflammatory Activity of Five Medicinal Mushrooms of the Genus *Pleurotus*

**DOI:** 10.3390/antiox11081569

**Published:** 2022-08-13

**Authors:** Jan Stastny, Petr Marsik, Jan Tauchen, Matej Bozik, Anna Mascellani, Jaroslav Havlik, Premysl Landa, Ivan Jablonsky, Jakub Treml, Petra Herczogova, Roman Bleha, Andriy Synytsya, Pavel Kloucek

**Affiliations:** 1 Department of Food Science, Faculty of Agrobiology, Food and Natural Resources, The Czech University of Life Sciences Prague, Kamýcká 129, 165 21 Prague, Czech Republic; 2 Laboratory of Plant Biotechnologies, Institute of Experimental Botany of the Czech Academy of Sciences, Rozvojová 313, 165 02 Prague, Czech Republic; 3 Department of Horticulture, Faculty of Agrobiology, Food and Natural Resources, The Czech University of Life Sciences Prague, Kamýcká 129, 165 21 Prague, Czech Republic; 4 Department of Molecular Pharmacy, Faculty of Pharmacy, Masaryk University, Palackého Třída 1946/1, 612 00 Brno, Czech Republic; 5 Department of Carbohydrates and Cereals, Faculty of Food and Biochemical Technology, University of Chemistry and Technology, Technická 5, 166 28 Prague, Czech Republic

**Keywords:** bioactivity, secondary metabolites, oyster mushroom, basidiomycetes, pink oyster mushroom, cyclooxygenase-2, immunomodulatory effect, radical scavenging effect, inflammation, Indian oyster mushroom

## Abstract

Within the group of higher fungi, edible medicinal mushrooms have a long history of being used as food and in folk medicine. These species contain biologically active substances with many potential beneficial effects on human health. The *Pleurotus* genus is representative of medicinal mushrooms because *Pleurotus ostreatus* is one of the most commonly cultivated culinary mushrooms. In our study, we focused on lesser-known species in the genus *Pleurotus* and measured their antioxidant and anti-inflammatory activity. We prepared extracts of the mushrooms and analyzed them using HPLC−HRMS, GC−MS, and ^1^H-NMR. Significant differences in biological activities were found among the *Pleurotus* spp. extracts. A MeOH extract of *P. flabellatus* was the most active as a radical scavenger with the highest ORAC, while a chloroform extract had significant anti-inflammatory COX-2 activity. The 80% MeOH extract of *P. flabellatus* contained the highest amounts of ergosterol, ergothioneine, and mannitol. The 80% MeOH extract of *P. ostreatus* Florida was the most active in the NF-κB inhibition assay and had the highest content of β-glucans (43.3% by dry weight). Given the antioxidant and anti-inflammatory properties of *P. flabellatus*, the potential therapeutic usefulness of this species is worth evaluating through in-depth investigations and confirmation by clinical trials.

## 1. Introduction

Edible mushrooms have been a significant part of human culture since ancient times [1] and those with medicinal properties have had a long tradition in the treatment of various human diseases [2]. In addition to their high content of micro and macronutrients [1], mushrooms also produce a wide array of secondary metabolites including biologically active compounds [2]. Out of the approximately 14,000 known mushroom species, about 700 are considered pharmacologically active [3]. Some of these mushroom species are consumed directly or are employed as food supplements and functional foods [4]. In view of their remarkable usefulness as both a food and a medicine, the new term, ‘mushroom nutraceutical’, was introduced to describe these edible medicinal mushrooms [5].

The genus *Pleurotus* includes types of edible, wood-decaying mushroom. Various *Pleurotus* species have been employed throughout history in medicine [6]; for example, they have bene used to strengthen joints, tendons, and muscles, improve cardiovascular health [7], and stimulate the immune system. Currently, there are 207 classified species in the genus *Pleurotus* [8]. The oyster mushroom, *P. ostreatus* (Fr.) *P. Kumm*, is one of the most economically significant species [9] and the second most important commercially cultivated mushroom [10]. It has been proven in numerous studies that *P. ostreatus* offers many health-promoting benefits such as anticancer, antioxidant, antitumor, antiviral, antibacterial, antidiabetic, and anti-hypercholesteremic activities. One of the most studied biological effects is the modulation of the immune system response, which involves different anti-inflammatory mechanisms [11]. There are several other *Pleurotus* species that are less common in the edible mushroom growing industry [12], but still possess some bioactive properties. The pink oyster mushroom (*P. flabellatus)* and the Indian oyster mushroom (*P. pulmonarius*) were reported to have antioxidant, anti-inflammatory, and antimicrobial activities as well [13,14,15,16,17,18,19,20,21,22,23,24].

The fruiting body of *P. ostreatus* contains approximately one hundred different compounds [9], including polysaccharide-proteins, polysaccharide-peptides, proteoglycans, functional proteins, and polysaccharides [25]. Specific proteoglycans from *P. ostreatus* have been found to have anticancer, immunomodulatory [26], and antioxidant activity [27]. The mushroom cell walls are rich in non-starch polysaccharides, of which β-D-glucans are the most interesting functional components [9] because they are the most common natural macrophage activators [26]. The α- and β-glucans and high molecular weight polysaccharides were reported to increase the production of cytokines by dendritic cells, activate natural killer cells, and increase the production of macrophages [11]. Besides the well-studied β-D-glucans, *P. ostreatus* contains other compounds, e.g., phenolic compounds like protocatechuic acid, gallic acid, homogentisic acid, rutin, myricetin, chrysin, naringin, α- and γ-tocopherol, ascorbic acid, β-carotene [9], cinnamic acid, p-hydroxybenzoic acid [28], and ergothioneine, many of which are potent antioxidants abundant in *P. ostreatus*; these are also found in other *Pleurotus* species [29].

Some other species or varieties of *Pleurotus* that are less frequently employed in the mushroom cultivation industry and have not been covered by local legislation differ in content and type of bioactive metabolites but may contain novel compounds not found in the commercially cultivated varieties of *P. ostreatus* [30]. Therefore, based on these facts, this study focused on the exploration of the less common species and varieties of the *Pleurotus* genus. Our major assumption was that because the various species and varieties of the genus differ extensively in the type and abundance of their constituents, they are likely to possess some novel biological activities.

## 2. Materials and Methods

### 2.1. Materials

#### 2.1.1. Samples and Cultivation

The following species and varieties were chosen from the collection of the Research Institute of Crop Production in Prague: *P. flabellatus* 5013, *P. pulmonarius* KZ50, *P. opuntiae* 5012, *P. ostreatus* Sylvan Ivory, and *P. ostreatus* 5175 Florida. *Pleurotus* spp. mushrooms were cultivated and harvested by the Department of Horticulture at CZU Prague on wheat straw pellets substrate. Two thousand five hundred g of substrate was pasteurized (90 °C for 24 h), moistened with water (67–69% humidity), and placed in polypropylene bags. Three percent grain spawn from selected *Pleurotus* samples was added into the cooled substrate. The samples were mixed thoroughly with the substrate and cultivated at 24 °C for 21 days. The samples were then switched to 17 °C in a relative humidity of 85–90% and a light of 1000 lux for the next 21 days and then harvested.

#### 2.1.2. Sample Preparation

The harvested mushrooms were freeze dried for three days, ground, and stored at room temperature in a dark place. A modified procedurewas used for sample extraction [31]. The sample (one gram) was extracted separately with (a) 12 mL of 80% methanol (80% MeOH) and (b) 10 mL of chloroform (CHL) plus 1 mL of distilled water for 30 min on an orbital shaker at 210 RPM. The extract was sonicated for 1 min at room temperature (Sonorex Digitec DT 255 H, 160/640 W, Bandelin, Berlin, Germany) and centrifuged at 24,400× *g* for 10 min at room temperature (Rotanta 460R, Hettich, Germany). The supernatant was transferred into an evaporation flask and the rest matrix was re-extracted using the same procedure. The supernatants were mixed and evaporated at 30 °C on a rotary evaporator (Büchi AG, Flawil, Switzerland). After evaporation, the flask containing the evaporated extracts was weighed and the extract were resuspended with the corresponding extraction solvent: (a) 10 mL of 80% MeOH or (b) 10 mL CHL. One mL of resuspended extract was collected for HPLC−HRMS analysis; the rest of the extract was evaporated, and then the flask was weighed again. These residual extracts were resuspended in dimethyl sulfoxide (DMSO) and collected in order to assay anti-inflammatory and antioxidant activities. For GC−MS analysis, the procedure was the same as the previous CHL procedure but the extraction solvent was hexane:diethyl ether (3:1, *v*/*v*) and the final resuspending volume was 2 mL. All extracts were prepared in triplicate and stored at −18 °C. For ^1^H-NMR spectroscopy, the extraction was performed based on a modified protocol proposed by Kim, Choi, and Verpoorte (2010) [32]. Each 50.0 mg of dried sample was extracted with 600 μL of 80% MeOH for 30 min with shaking. The extracts were sonicated for 5 min at room temperature and centrifuged at 24,400× *g* for 10 min to separate the extract from the matrix. After collecting the supernatants, the extraction process was repeated with 600 µL of the same solution. Both supernatants were mixed and evaporated using a stream of nitrogen gas. The residue was dissolved in 400 μL of deuterated methanol-D_4_ and 400 μL of KH_2_PO_4_ buffer in deuterated water (pH 6.0) containing 0.1% TSP-D_4_ (trimethylsilylpropionic acid, sodium salt-D4, *wt*/*wt*). The mixture was centrifuged at 24,400× *g* for 10 min at room temperature. Supernatants (600 μL) were transferred to 5 mm NMR tubes and subjected to ^1^H-NMR analyses (see Section 2.2.3).

### 2.2. Chemical Analysis

#### 2.2.1. Liquid Chromatography−Mass Spectrometry (LC−MS) Analysis

A non-targeted screening analysis of the crude extracts (80% MeOH) from the five *Pleurotus* species was performed using the LC-HRMS system consisting of the Ultimate 3000 UPLC chromatograph Thermo Fisher Scientific (Waltham, MA, USA) with a Q-TOF high-resolution mass spectrometer (Impact II, Bruker Daltonic, Bremen, Germany). An Acclaim RS-LC 120 C18 column (2 µm, 2.1 × 100 mm, Thermo Scientific, Waltham, MA, USA) was used for chromatographic separation. The column temperature was set at 35 °C. Two types of polar phases (A1—5 mM ammonium formate (COONH_4_) and A2—0.1% formic acid (HCOOH) in water) were chosen as the mobile phase due to the differing ionization properties of the molecules. Methanol (MeOH) was used as the organic phase (B). In order to ensure the widest possible range of polarity of the analytes contained in the extracts, a gradient ranging from 2% to 100% organic phase (MeOH) over 26 min and then isocratic for 10 min with 100% MeOH, was used for analysis. Each sample was analyzed with both mobile phase systems (A1/B, A2/B). The flow rate of the mobile phase was 0.25 mL/min and the injection volume of the sample was 5 μL. The *m*/*z* range of the monitored masses was between 60 and 1500. For the non-target analysis, an electrospray ionization (ESI) in both positive and negative mode data was applied. The data were collected in ddMS^2^ mode in order to contain fragmentation spectra of the most important ions for later identification.

The content of ergosterol was determined using the same LC−MS system with APCI ionization in positive mode. The mobile phase consisted of 0.2% formic acid (polar phase A) and methanol (organic phase B). An elution gradient was used: for the first minute the mobile phase was kept on 60% B, then the percentage of B grew to 100% in 6 min, and remained on this level for the next 10 min. Then, the level of B decreased to the starting conditions (60% B) and was kept at this level for 5 min to achieve system equilibration. The same chromatographic column that was used in the non-target analysis was used. The injection volume of samples was 5 μL. The flow rate of the mobile phase was set to 0.35 mL/min and the column temperature was set to 35 °C. Ergosterol was identified by the commercially available standard (Pharmaceutical Secondary Standard; Certified Reference Material, Sigma-Aldrich, St. Louis, MO, USA). Detailed mass spectrometry measurement parameters for both ion source analyses are shown in Table S1. The identification of ergosterol was based on retention time and the exact mass of the ion [M + H]^+^ = 397.3470, which was also used as a quantification ion, and [M-H_2_O]^+^ = 379.3365, which was used as a confirmation ion. The following values were calculated for ergosterol: LOD = 121.8 ng/mL, LOQ = 406.1 ng/mL, and a precision (RSD) of 2.34%. Ten points of the concentration range 20–2500 ng/mL were used for calibration curve construction and the R^2^ of the curve was 0.9991. A carryover effect was monitored by measuring solvent blanks between the samples. The quantification of analytes was performed using TASQ 2.2 software (Bruker Daltonik, Bremen, Germany).

In the non-target analysis, the data acquisition and the first step of data processing was performed by Otof Control 5.2, Bruker Compass Hy Star 5.1 and DataAnalysis 5.2 software (all Bruker Daltonik, Bremen, Germany). The results were then processed using XCMS (Scripps Research) and Mzmine software (authors: Matej Orešič, Mikko Katajamaa) and based on these outputs, sets of difference signals (features) were identified for each type of crude extract. The total number of identified features (about 120,000) was subsequently reduced to the resulting several hundred potential metabolites by applying criteria like intensity, peak shape, *m*/*z* and retention time, and exclusion of isotopic ions and adducts. The reduced dataset (5000 features) with the highest importance was normalized (Pareto scaling), cleaned using principal component analysis (PCA) in the online free software MetaboAnalyst (v 5.0, Xia Lab, Ste. Anne de Bellevue, QC, Canada) by selection of the most important features responsible for the biggest differences among samples. The final number of features was then reduced to 330 by removing in-source fragment ions and other artifacts.

#### 2.2.2. Gas Chromatography Coupled to Mass Spectrometry (GC−MS) Analysis

For GC−MS, a modified sample preparation protocol by Pedneault et al. (2007) was used [33]. GC Agilent Technologies 7890A, MS 5975C (Palo Alto, CA, USA) was used for the analysis of non-polar compounds extracted with hexane:diethyl ether (3:1, *v*/*v*). One µL of extract was injected in split mode (1:12) into a system equipped with an Agilent J&W DB-5MS column (Palo Alto, CA, USA). The separation was performed using a temperature gradient program, starting at a temperature of 60 °C, which then increased to 280 °C in 3 °C increments. Mass spectra were collected in TIC mode.

#### 2.2.3. Proton Nuclear Magnetic Resonance (^1^H-NMR) Analysis

The ^1^H-NMR parameter for spectral acquisition was used according to the protocol by Mascellani et al. (2021) [34]. All spectra were recorded at 298 K (25 °C) on a Bruker Avance III HD spectrometer equipped with a broadband fluorine observation (BBFO) SmartProbe™ with z-axis gradients (Bruker BioSpin GmbH, Rheinstetten, Germany), operating at a ^1^H-NMR frequency of 500.18 MHz. The spectrometer transmitter was locked to deuterated MeOH and all spectra were recorded with the Bruker pulse sequence ‘noesypr1d’ for presaturation of the water signal at 4.704 ppm. Each sample was collected in 64 k data points after 128 scans and 4 dummy scans using a spectral width of 8000 Hz. The receiver gain was set to 18, the relaxation delay was 1 s, the acquisition time was 4 s, and the mixing time was 0.1 s. The free induction decay was multiplied by 0.3 Hz (line broadening) before Fourier transformation. TSP was used to calibrate to 0.0 ppm. The acquired ^1^H-NMR spectra were phased and corrected for baseline using Chenomx NMR suite 9 software professional edition (Chenomx Inc., Edmonton, AB, Canada). The assignment of signals was performed using spiked samples, in-house, and built-in databases. The spiking for ergothioneine was performed using the commercially available standard (purity ≥ 98.0%, Sigma-Aldrich, St. Louis, MO, USA) to confirm the annotation.

### 2.3. Antioxidant Activity

#### 2.3.1. Radical Scavenging Assay Using 2,2-Diphenyl-1-picrylhydrazyl (DPPH)

The ability of the tested samples to inhibit DPPH (2,2-diphenyl-1-picrylhydrazyl; Sigma-Aldrich, St. Louis, MO, USA) radicals was determined using the method described by Sharma and Bhat (2009) [35] with modification. Initially, two-fold serial dilutions of each sample were prepared in analytical grade MeOH (VWR, Radnor, PA, USA) in 96-well microtiter plates. Subsequently, 100 μL of freshly prepared 0.25 mM DPPH in MeOH was added to each well and mixed with the samples, creating a concentration range of 512 to 8 μg/mL. Trolox (6-hydroxy-2,5,7,8-tetramethylchroman-2-carboxylic acid; Sigma-Aldrich, St. Louis, MO, USA) was used as a positive control at concentrations of 64, 32, 16, 8, 4, 2, and 1 μg/mL, with MeOH used as a blank. The absorbance was measured at 517 nm using a Synergy H1 multi-mode reader (BioTek, Winooski, Winooski, VT, USA). Results were expressed as half-maximal inhibitory concentrations (IC_50_ in μg/mL) and Trolox equivalents (mg TE/g extract).

#### 2.3.2. Oxygen Radical Absorbance Capacity (ORAC) Assay

The sample’s ability to retard the AAPH-induced oxidant decay of fluorescein (FL) was measured by the method of Ou and Hampsch-Woodill (2001) [36] with modification. All samples and reagents were prepared and diluted in 75 mM phosphate buffer (pH adjusted to 7.0 with HCl). Inorganic acids and salts used for buffer preparation included monopotassium phosphate (KH_2_PO_4_, Lach-ner, Neratovice, Czech Republic), dipotassium phosphate (K_2_HPO_4_, Sigma-Aldrich, St. Louis, MO, USA), and hydrochloric acid (Penta, Czech Republic). The outer wells of black absorbance 96-well microtiter plates were filled with 200 μL of distilled water in order to provide better thermal mass stability, as suggested by Held (2005) [37]. Afterwards, 25 μL of each sample was transferred to the plates, diluted with 150 μL of fluorescein (54 nmol/L) (Sigma-Aldrich, St. Louis, MO, USA), and incubated at 37 °C for 10 min. To initiate the reaction, 25 μL of 153 mmol/L AAPH (2,2′-azobis(2-amidinopropane) dihydrochloride, Sigma-Aldrich, St. Louis, MO, USA) was added to each well. Fluorescence decay was measured at one-minute intervals for two hours with excitation and emission wavelengths set at 494 and 519 nm. Trolox at 8, 4, 2, 1, and 0.5 μg/mL was used as a positive control with the buffer used as a blank. Samples were tested at a final concentration of 1024 μg/mL. The ORAC values were calculated as the area under the curve (AUC) values and compared with the calibration curve of Trolox, as previously suggested by Ou and Hampsch-Woodill (2001) [36]. Results were expressed as Trolox equivalents (mg TE/g extract).

### 2.4. COX-2 Anti-Inflammatory Activity Assay

Human recombinant cyclooxygenase-2 (COX-2; Sigma-Aldrich, St. Louis, MO, USA) was used in an in-vitro enzymatic assay. COX-2 (0.5 unit/reaction) was added to 180 µL of 100 mM Tris buffer (pH 8.00) containing 5 μM hematin (porcine), 18 mM (L)-(−)-epinephrine, and 50 μM Na_2_EDTA. Mushroom extracts in 80% MeOH were evaporated and dissolved in DMSO (VWR, Radnor, PA, USA). The final concentration of the extracts in the reaction was 10 μg/mL. Pure DMSO was used as a blank and (S)-(+)-ibuprofen (Sigma-Aldrich, St. Louis, MO, USA) was used as a reference inhibitor (positive control). Aliquots of 10 μL of extract were incubated with the reaction mixture for 5 min at room temperature. The reaction was then initiated by the addition of 5 µL of 10 μM arachidonic acid and incubated for 20 min at 37 °C. The reaction was stopped by the addition of 20 µL of 10% formic acid (*v*/*v*). The inhibitory activity was calculated as the percentage inhibition of prostaglandin E_2_ (PGE_2_) production compared to the blank. The concentration of PGE_2_ was quantified using a prostaglandin E_2_ ELISA kit (Enzo Life Sciences, Farmingdale, NY, USA). Samples were diluted (1:15) and incubated according to the manufacturer’s instructions. Absorbance was measured at 405 nm using a Tecan Infinite M200 microplate reader (Tecan Group, Männedorf, Switzerland). The results were expressed as the percent inhibition of the sample compared to the blank.

### 2.5. Cell-Based Assays

The THP-1-XBlue™-MD2-CD14 cell line was purchased from Invivogen (San Diego, CA, USA) and was cultured as reported previously by Hosek et al. (2011) [38]. In more detail, the cells were cultivated at 37 °C in RPMI 1640 medium supplemented with 2 mM L-glutamine, 10% FBS, 100 U/mL penicillin, and 100 μg/mL streptomycin in a humidified atmosphere containing 5% CO_2_. The WST-1 assay was used to test the effects of extracts on the viability of using the THP-1-XBlue™-MD2-CD14 cells according to the manufacturer’s instructions. The antiproliferative activity of the test extracts from *P. flabellatus* 5013, *P. opuntiae* 5012, *P. ostreatus* 5175 Florida, and *P. pulmonarius* KZ50 was tested at five concentrations ranging from 0.25 to 20 μg/mL. Based on these results, the non-toxic extract concentration of 10 µg/mL was used for subsequent cell-based assays.

#### 2.5.1. Cellular Antioxidant Activity (CAA) Assay

The antioxidant activity of test extracts was measured using the method of Wolfe and Liu (2007) [39] with some modifications, as reported by Malanik et al. (2020) [40]. THP-1-XBlue™-MD2-CD14 cells were pre-incubated for 1 h in a serum-free RPMI 1640 medium containing 25 μM 2′,7′-dichlorodihydrofluorescein-diacetate (DCFH_2_-DA; Sigma-Aldrich, St. Louis, MO, USA) dissolved in DMSO [the final concentration of DMSO in the medium was 0.1% (*v*/*v*)] at 37 °C. After that, the cells were centrifuged, washed with phosphate-buffered saline, re-suspended in serum-free RPMI 1640 medium, and placed into 96-well plates in triplicates—60,000 cells/well. The cells were then incubated with the extracts for 1 h and after that, 2,2′-azobis(2-methylpropionamidine) dihydrochloride (AAPH; Sigma-Aldrich, St. Louis, MO, USA) was added (at a final concentration of 600 µM) to induce the generation of ROS. The plate was immediately placed into a FLUOstar Omega microplate reader (BMG Labtech) tempered at 37 °C. The level of oxidized fluorescent 2′,7′-dichlorfluorescein (DCF) was measured every 5 min for 1 h (excitation wavelength at 485 nm; emission at 538 nm). Each plate included triplicate control and blank wells: control wells contained cells treated with DCFH-DA and AAPH; blank wells contained cells treated with the dye and serum-free RPMI 1640 medium without AAPH. Quercetin (Koch-Light Laboratories, Haverhill, UK) was used as a positive control at the same concentration as the test compounds at a concentration of 10 μg/mL. The solvent (80% MeOH) was used as the negative control (NC).

After the blank was subtracted from the fluorescence readings, the area under the curve of fluorescence versus time was integrated to calculate the CAA values of the test compounds: CAA unit = 100 − (∫SA/∫CA) × 100, where ∫SA is the integrated area under the sample fluorescence versus time curve and ∫CA is the integrated area obtained from the control curve.

#### 2.5.2. Detection of the Activation of NF-κB/AP-1

The anti-inflammatory activity of the extracts was also investigated in terms of their effect on NF-κB/AP-1 signaling in lipopolysaccharide (LPS)-stimulated THP-1-XBlue™-MD2-CD14 cells (Invivogen, San Diego, CA, USA). The cell line expresses an NF-κB/AP-1-inducible secreted embryonic alkaline phosphatase (SEAP) reporter gene. The cells were incubated for 24 h with test extracts dissolved in 80% MeOH at a concentration of 10 µg/mL. Solvent alone was used as a negative control (NC) and 10 μM prednisone was used as a positive control (PC).

### 2.6. Determination of the Content of Glucans

The “MUSHROOM and YEAST β-GLUCAN” K-YBGL 07/11 analytical set (Megazyme International, Wicklow, Ireland) was used for the determination of the total α- and β-glucans [41]. The assay determines the difference between the glucose content after total acidic hydrolysis of glucans and specific enzymatic hydrolysis of starch-like α-glucans. Samples were solubilized in concentrated hydrochloric acid (37%; 10 N) and then hydrolyzed with 1.3 mol/L hydrochloric acid at 100 °C for 2 h. Total hydrolysis was completed via incubation with a mixture of exo-(1–3)-β-glucanase and β-glucosidase. Starch-like α-glucans were solubilized in 2 mol/L potassium hydroxide and the mixture was neutralized with an excess of 1.2 mol/L sodium acetate buffer (pH 3.8). The dissolved glucans were then hydrolyzed with amyloglucosidase. The β-glucan, or non-starch glucan, content was calculated as the difference between the glucose content after total acidic hydrolysis of glucans and specific enzymatic hydrolysis of α-glucans. All measurements were made in triplicate.

### 2.7. Statistical Analysis

Statistical analyses were carried out using Statistica (TIBCO Software, CA, USA) and MetaboAnalyst v. 5.0 (Xia Lab, Ste. Anne de Bellevue, QC, Canada). The data are presented as means ± SD. To determine the significance of sample differences, one-way ANOVA, followed by a post-hoc Tukey test (α = 0.01) was used. For cell-based assays, the statistical analyses were carried out using IBM SPSS for Windows, software version 26.0 (Armonk, NY, USA). The data were graphed as a mean ± SD. Comparisons between groups were made using a Kruskal–Wallis test, followed by pair-wise comparison with Bonferroni correction.

## 3. Results and Discussion

### 3.1. Extraction Yield of Mushroom Extracts

Table 1 shows the extraction yields from mushroom samples. There was a significant difference in the extraction yield between the extraction solvents. The yield of 80% MeOH samples ranged between 28.4 and 32.9% and was higher than the yield of CHL extracts, which ranged from 2.5–4.5%.

The difference may have been caused by the low content of lipids and nonpolar compounds in the mushroom samples. Eighty percent MeOH has a high extraction power for a broad spectrum of the polar or slightly nonpolar compounds present in mushrooms. According to Alam et al. [42], dried mushrooms of three *Pleurotus* species were determined to contain 4.4–4.6% lipids, 38.8–42.8% carbohydrates, 20.6–24.6% proteins, 8.3–9.4% ash, and 22.9–24.3% fiber. According to Chaiharn et al. [43], the extraction yield of two *P. ostreatus* samples was 16.00% and 22.25% for water and 6% and 10% for MeOH.

### 3.2. LC−MS Analysis of Mushroom Extracts

Preliminary identification based on the screening data obtained from chloroform extracts indicated the presence of a number of lipids such as phospholipids and acyl-glycerol- and steroid-type compounds. Higher molecular weight compounds, such as oligosaccharides, peptides, and their conjugates with smaller molecules, were found in 80% MeOH extracts, as indicated by the collected MS^2^ spectra. Untargeted HPLC−HRMS analysis by PCA score plot based on 330 of the most important features (Figure S1) showed specific differences between *P. flabellatus* and other samples, which was confirmed by hierarchical clustering (Figure S2). Subsequent tentative identification of the most important features using MS^2^ analysis showed that most of them were related to higher molecular structures, especially oligosaccharides and peptides.

An example of an identified low molecular weight metabolite with possible biological activity in these extracts was ergothioneine, whose identity was also confirmed by the fragment spectrum (Figure S3). Another abundant compound identified in the analysis was ergosterol (Table 2).

The highest ergosterol content was found in *P. flabellatus* 5013 and the lowest content was in *P. ostreatus* Sylvan Ivory. The ergosterol level in selected mushroom samples ranged from 214.5–163.7 mg/kg of d.w. powder and 538.5–805.3 µg/g of dry extract.

### 3.3. GC−MS Analysis of Mushroom Extracts

The predominant compound in all measured extracts was ergosterol, a precursor of vitamin D_2_. The other most abundant compounds were isomers of 2-butyl-2-octanol, dodecanol, and 2-hexyl-1-octanol derivatives, which are common in mushrooms [44]. The *P. ostreatus* 5175 Florida showed the greatest difference among the samples because of the presence of octadecadienoic acid derivatives (Figure S4).

### 3.4. NMR Analysis of Mushroom Extracts

Table 3 shows the results of the ^1^H-NMR spectroscopy of selected analytes. The most abundant were the saccharides trehalose and mannitol, followed by specific amino acids, organic acids, and amines including choline and ergothioneine. The highest content of ergothioneine was detected in *P. flabellatus* 5013. In general, the ergothioneine content was higher in lesser-known species compared to *P. ostreatus* samples. The most abundant substance was trehalose (105.0–347.7 mg/g of dry extract); however, in *P. flabellatus* 5013, the mannitol content was higher than trehalose. The content of mannitol ranged from 18.14 to 144.3 mg/g of the extract and the lowest mannitol level was found in *P. ostreatus* 5175 Florida. Since all tested mushrooms were cultivated in similar conditions, this variability may be mainly caused by genetic differences and subsequent changes in metabolic pathways. According to Lin et al.’s [45] phylogenetic analysis of *Pleurotus* species, *P. ostreatus* and *P. flabellatus* are the most distinct among the tested species, which is in agreement with the results of our metabolomic and bioactivity investigations, wherein usually these two species are the most distinct.

### 3.5. Content of Glucans

The total glucan content (Table 4) ranged from 35.8 ± 0.3% to 49.0 ± 0.3% of d.w. and *P. opuntiae* 5012 had the highest content of α-glucans (4.5–9.8%). The range of β-glucan content was 31.53 ± 0.6% to 43.3 ± 0.6% *w*/*w* and *P. ostreatus* 5175 Florida had the highest β-glucan content of all the samples (43.3 ± 0.6%, *w*/*w*). According to Avni et al. [46], the total glucan content ranged from 20.25 ± 0.52% to 48.27 ± 0.68% in *Pleurotus* spp. and the α-glucan content was 0.47 ± 0.02% to 4.57 ± 0.06%. Lam and Okello [47] determined that the total glucan content of *P. ostreatus* PL 132 was 27.1% (*w*/*w*) and the β-glucan content was 23.9% (*w*/*w*). The differences in glucan levels may have been caused by genetic factors.

### 3.6. Antioxidant Activity

There was a difference in ORAC and DPPH antioxidant activity between the different solvent extracts (Table 5). The IC_50_ of CHL extracts was <0.68 mg of TE (Trolox equivalent)/g extract, which is considered very low (data not shown). The activity of 80% MeOH extracts ranged from 4.3 to 24.9 mg of TE/g extract in the DPPH assay and 21.7 to 63.9 mg of TE/g extract in the ORAC assay. *P. flabellatus* 5013 was the most active species in both assays.

The advantage of CAA assays over ORAC and DPPH assays is the involvement of the cellular environment in the activity [48]; however, our results in the CAA assay did not confirm the promising activity of the *P. flabellatus* 5013 extract. None of the tested extracts had significantly higher activity than the quercetin control. The observed discrepancy might be explained by the different mechanisms tested in chemical-based antioxidant assays (e.g., ORAC and DPPH) and CAA, a cell-based assay. For an extract to be active in the CAA assay, first, the cellular membrane must be crossed by the active constituents. Secondly, the antioxidants must then scavenge various types of peroxyl radicals and other ROS formed in a cellular environment, which are obviously different from those formed in ORAC or DPPH assays. The tested extracts proved inactive in either one of these two aspects or both [48].

Bakir et al. [49] measured the DPPH antioxidant activity in *P. ostreatus* stored for 24 h at 20 °C and obtained an IC_50_ of 321 μg/mL, while a sample stored at −40 °C had significantly decreased antioxidant activity with an IC_50_ of 3486 μg/mL. The IC_50_ range in our study was 200.4 to 1134.0 μg/mL. We used three-day lyophilized mushroom samples, stored frozen and our IC_50_ results are similar to those of Bakir et al. [49]. The antioxidant activity of mushroom extract may be linked to ergothioneine (ERG), a water-soluble thiol derivative of histidine, which was previously shown to be associated with antioxidant activity in vitro and in vivo [50]. Many studies confirmed the presence of ergothioneine in various *Pleurotus* species [29,51,52,53].

According to Halliwell, Cheah, and Tang (2018), the ERG content in food is quite low, yet the human body can accumulate it efficiently. ERG is transported in the blood via a specific transporter (OTCN1) and ERG levels appear to decline during neurodegenerative and cardiovascular disorders, which are associated with oxidative stress. This suggests that ERG may play an important role as a dietary antioxidant. The ERG content in various foods is variable. *P. eryngi*, for example, was found to contain 541.7 mg of ERG per kg dry weight and in other mushrooms, it ranged from 5.8 to 1812.4 mg/kg d.w [50]. The ERG content of *P. cintronopileatus* was 3.94 mg/g d.w. compared to 1.21 mg/g d.w. in *P. ostreatus* [52], which indicated that some lesser-known species of *Pleurotus* could contain a higher concentration than *P. ostreatus*. A few non-mushroom foods such as tempeh have a high ERG content (2011.3 mg/kg), but foods like rice, milk, nuts, beans, spices, and vegetables have significantly lower ERG levels at about 49.2 mg/kg d.w. Only fungi are considered to have the necessary biosynthetic apparatuses for ERG biosynthesis and its presence in tempeh is perhaps the result of culturing with *Rhizopus* fungi [50]. Dubost et al. [54] determined that *P. ostreatus* contained 2010 mg of ERG per kg d.w. and *P. eryngii* had 1720 mg ERG/kg d.w., which is comparable to our results.

Tsiapali et al. [55] showed that polysaccharides, such as glucans, have only weak in vitro free radical scavenging ability and may actually stimulate free radical activity in murine macrophages, increasing the level of reactive oxygen species associated with immunomodulation. Trehalose, a disaccharide found in our samples in large quantities, was also proven to decrease oxidative stress in vivo by enhancing the expression of antioxidant genes [56]. According to Radbakhsh et al. [57], trehalose was effective in increasing the total antioxidant capacity and thiol groups in serum in an experimental type 2 diabetes rat model. J.-H. Liu et al. [58] claimed that mannitol acted like a free-radical scavenging agent in vivo against peroxyl radicals and Pelle et al. [59] showed that mannitol was able to protect DNA against UV damage in vivo but was not effective against oxygen radicals, as determined by increased malondialdehyde as a marker level in vivo. Results published by Meza-Menchaca et al. [60] demonstrated that ergosterol peroxide may also be linked to antioxidant activity. The analysis of antioxidant activity in the presence of 80% MeOH extract constituents showed a high correlation of ERG content with IC_50_ (R = −0.71%) and nicotinate content (−0.90%) (the lower IC_50_ means higher antioxidant activity). Nicotinate or niacin (vitamin B_3_) is part of an important redox agent in the NAD(P)^+^/H system [61] and, therefore, has the ability to prevent oxidative stress [62,63] and act as an anti-inflammatory agent [63].

### 3.7. Anti-Inflammatory Activity

In the COX-2 inhibitory assay, the non-polar CHL extracts were more active than the 80% MeOH extracts (Table 6), which were virtually inactive and did not even reach the 50% inhibition level. The CHL extract activity was 43.6% to 85.0% of the control. The most active extract in the COX-2 assay was from *P. flabellatus* 5013. In the NF-κB/AP-1 inhibition assay, the extract activity ranged from 72.7% to 83.4% of the NC, while the inhibitory effect of prednisone (PC) was 79.2 ± 8.0%. The most active sample was *P. pulmonarius* KZ50. Each assay involves a different mechanism, so the results are not fully comparable.

As reported in previous studies, ergosterol may also be linked to anti-inflammatory and immunomodulatory effects. Ergosterol decreased p-NF-κB and COX-2 expression in a mouse model of diabetic nephropathy [64]. Ergosterol is one of the most abundant metabolites in many fungal species and it was detected in the mushroom extracts in our study as well as in other studies [65,66,67,68]. Previous studies suggested that ergosterol, a provitamin form of vitamin D_2_ whose mechanism of effect is unclear, is beneficial to human health by decreasing the risk of inflammatory disease. According to clinical studies, vitamin D deficiency is associated with inflammatory health risks [69].

The work of Du et al. [70] dealt with different types of glucans, including those that possess immunomodulatory and anti-inflammatory effects, mediated through cytokines such as TNF-α. The extract from *P. ostreatus* 5175 Florida was determined to contain the highest amount of total glucans and β-glucans and this may explain why it was the most active in inhibiting NF-κB/AP-1 activity. According to Deo et al. [71], an anti-inflammatory assay of *P. ostreatus* extract showed over 90% inhibition of TNF-α in the LPS-stimulated mouse macrophage cell 264.7.

Trehalose, which was also assayed in our study, was found to suppress NF-κB pathway activation in cultured cells [72]. The extract of *P. ostreatus* 5175 Florida was most effective at inhibiting NF-κB (72.2 ± 7.7% of NC), while the lowest activity was recorded for *P. flabellatus* 5013 extract, which correlated with the lowest measured trehalose level. However, it was reported that increasing the dietary level of trehalose by adding it to food may increase the risk of severe *Clostridium* infection in the gut [73].

Jedinak et al. [11] presented evidence that the *P. ostreatus* Florida extract had anti-inflammatory activity as determined by an NF-κB/AP-1 assay in murine splenocytes and it may be effective as a dietary supplement for the reduction of inflammation. In the study, mushroom extracts were added to LPS-activated RAW264.7 mouse macrophages and an extract concentration of 100 µg/mL decreased TNF-α levels from 15.4 to 5.87 ng/mL, IL-6 from 16.1 to 1.16 ng/mL, and IL-12 from 108.8 to 39.2 ng/mL compared controls without extract.

An in vivo study of Wistar rats using the carrageenan-induced paw edema model of inflammation compared the anti-inflammatory activity of dried powdered *P. ostreatus* with an acetone extract of the same mushroom. The powdered sample at doses of 250–1000 mg/kg exerted a significant inhibition of rat paw edema, but the effect was not dose-dependent. The acetone extract of *P. ostreatus* (500 mg/kg) showed a maximum 87% inhibition of late phase inflammation, and this was also not dose-dependent [74].

## 4. Conclusions

In our study, we examined the biological activities of several species of mushrooms of the *Pleurotus* genus, including lesser-known varieties, and attempted to identify the compounds that were responsible for the observed effects. There is evidence that the less common varieties and species may have greater potential for antioxidant and anti-inflammatory properties than the conventional varieties. In the measurement of the total and β-glucans, the highest content was found in *P. ostreatus*, but there was relatively little difference in the content among the other species. *P. flabellatus* 5013 contained the highest level of ergosterol and mannitol was the most abundant compound determined. The results of the in vitro assays were confirmed by in vivo assays. *Pleurotus flabellatus* 5013 extract was the most effective in the antioxidant assay, which may be linked to the higher measured level of ergothioneine. In other samples, the most abundant compound was trehalose. In antioxidant assays, the 80% MeOH extracts displayed activity, while the CHL extracts did not. These results may also be linked to the ergothioneine content, as confirmed by ^1^H-NMR and LC−MS analysis of the extracts. The COX-2 anti-inflammatory assay showed that CHL extracts were more active than 80% MeOH extracts. The ergosterol content determined in our study may be associated with this activity. The *P. ostreatus* 5175 Florida extract was the most active in the NF-κB inhibition assay, and this may be linked to the fact that it contained the highest total glucan content. On the other hand*, P. flabellatus* 5013, which had the highest antioxidant activity, was found to contain the lowest amount of total and β-glucans. A clinical trial to test the potential therapeutic effects of *Pleurotus* extracts ought to be conducted to confirm the results of this study.

## Figures and Tables

**Table 1:** Yield of *Pleurotus* mushroom extract with different extraction solvents.

Sample Name	Extraction Yield by Solvent (% *w*/*w* d.w.)	
	80% MeOH	CHL
*P. flabellatus* 5013	30.8 ± 2.4 ^a^	2.7 ± 0.2 ^b^
*P. pulmonarius* KZ50	30.4 ± 0.9 ^a^	2.5 ± 0.2 ^b^
*P. opuntiae* 5012	28.4 ± 0.6 ^a^	3.5 ± 0.3 ^c^
*P. ostreatus* Sylvan Ivory	30.2 ± 2.7 ^a^	4.5 ± 0.2 ^a^
*P. ostreatus* 5175 Florida	32.9 ± 2.5 ^a^	4.0 ± 0.4 ^a,c^

The a–c within a column means that significance was determined by one-way ANOVA followed by post-hoc Tukey test (*p* < 0.01).

**Table 2:** Ergosterol content of 80% MeOH extracts of *Pleurotus* species.

Sample	Ergosterol Content	
	mg/kg of d.w. Powder	µg/g of Dry Extract
*P. flabellatus* 5013	214.5 ± 8.1 ^b^	805.3 ± 30.2 ^b^
*P. pulmonarius* KZ50	166.3 ± 2.2 ^a^	629.7 ± 8.2 ^a^
*P. opuntiae* 5012	163.4 ± 13.1 ^a^	604.3 ± 48.3 ^a^
*P. ostreatus* Sylvan Ivory	163.7 ± 8.2 ^a^	538.5 ± 27.0 ^a^
*P. ostreatus* 5175 Florida	178.7 ± 3.1 ^a^	547.7 ± 9.3 ^a^

The a,b within a column means that significance was determined by one-way ANOVA followed by post-hoc Tukey test (*p* < 0.01).

**Table 3:** ^1^H-NMR spectroscopy of genus *Pleurotus* mushroom sample 80% MeOH extracts.

Compound	Sample				
	*P. flabellatus* 5013	*P. pulmonarius* KZ50	*P. opuntiae* 5012	*P. ostreatus* Sylvan Ivory	*P. ostreatus* 5175 Florida
	Content (µg/g of Dry Extract)				
2-Aminobutyrate	398.8 ± 12.7 ^a^	512.3 ± 53.9 ^c^	257.6 ± 7.2 ^b^	323.4 ± 13.5 ^a^	214.5 ± 14.9 ^b^
Acetate	439.6 ± 14.5 ^a^	956.3 ± 125.8 ^b^	452.4 ± 21.1 ^a^	612.1 ± 30.7 ^a,b^	661.1 ± 29.2 ^b^
Nicotinate	900.7 ± 72.3 ^b^	580 ± 70.1 ^a,c^	757.8 ± 53 ^a,b^	672.4 ± 53.1 ^a,b^	377.5 ± 29.5 ^c^
Tryptophan	1080.6 ± 111.1 ^b^	569.3 ± 70.4 ^c^	275.5 ± 31.4 ^a^	397.7 ± 29.9 ^a,c^	354.9 ± 19.1 ^a,c^
Valine	879.8 ± 10.3 ^a^	1264.4 ± 108.8 ^b^	761.4 ± 38.1 ^a^	785.9 ± 17.6 ^a^	584.6 ± 21.8 ^a^
	**Content (mg/g of dry extract)**				
Alanine	8.35 ± 0.89 ^a^	8.43 ± 0.45 ^a^	8.15 ± 0.30 ^a^	6.76 ± 0.12 ^a^	6.14 ± 0.25 ^a^
Aspartate	3.714 ± 0.055 ^a,b^	6.02 ± 0.44 ^b^	10.76 ± 0.35 ^c^	4.20 ± 0.13 ^a^	8.45 ± 0.33 ^c^
Choline	4.40 ± 0.17 ^b^	5.39 ± 0.23 ^a^	3.915 ± 0.089 ^b^	4.58 ± 0.17 ^a^	4.25 ± 0.12 ^a^
Ergothioneine	6.22 ± 0.47 ^b^	3.13 ± 0.21 ^a^	4.16 ± 0.13 ^c^	2.65 ± 0.14 ^a^	2.72 ± 0.15 ^a^
Fumarate	1.70 ± 0.27 ^b^	3.40 ± 0.24 ^d^	4.30 ± 0.12 ^c^	4.99 ± 0.22 ^a^	3.14 ± 0.12 ^c,d^
Glutamate	14.8 ± 1.3 ^a,b^	8.87 ± 0.81 ^c^	13.40 ± 1.3 ^a,b,c^	15.5 ± 1.9 ^a^	8.82 ± 0.44 ^b,c^
Glutamine	25.92 ± 0.95 ^b^	9.75 ± 0.16 ^c^	8.26 ± 0.38 ^c^	11.358 ± 0.085 ^a^	8.72 ± 0.36 ^a,c^
Mannitol	144.3 ± 4.6 ^b^	31.5 ± 3.1 ^a^	24.09 ± 0.88 ^a^	18.14 ± 0.53 ^a^	13.81 ± 0.61 ^a^
Phenylalanine	2.38 ± 0.26 ^b^	2.36 ± 0.17 ^b^	1.587 ± 0.054 ^a^	1.33 ± 0.11 ^a^	1.734 ± 0.094 ^a,b^
Succinate	1.763 ± 0.088 ^b^	6.00 ± 0.38 ^d^	26.769 ± 0.044 ^c^	3.10 ± 0.13 ^a^	2.451 ± 0.087 ^a,c^
Trehalose	105.0 ± 2.1 ^b^	318.7 ± 20.2 ^a^	281.6 ± 3.9 ^a^	270.7 ± 9.2 ^a^	347.7 ± 11.5 ^c^
Tyrosine	2.275 ± 0.052 ^b,c^	2.15 ± 0.14 ^b^	2.531 ± 0.088 ^c^	2.967 ± 0.079 ^a^	1.700 ± 0.044 ^b^

The a–d within a column means that significance was determined by one-way ANOVA followed by post-hoc Tukey test (*p* < 0.01).

**Table 4:** Content of glucans in *Pleurotus* mushroom extracts.

Sample Name	Content (% *w*/*w* of d.w.)		
	Total Glucans	α-Glucans	β-Glucans
*P. flabellatus* 5013	35.8 ± 0.3 ^a^	4.5 ± 0.4 ^a^	31.5 ± 0.6 ^a^
*P. pulmonarius* KZ50	41.4 ± 0.1 ^b^	6.5 ± 0.8 ^ab^	34.9 ± 0.9 ^a^
*P. opuntiae* 5012	44.4 ± 0.3 ^c^	9.8 ± 0.1 ^b^	34.5 ± 0.5 ^a^
*P. ostreatus* Sylvan Ivory	36.5 ± 0.1 ^a^	3.8 ± 1.2 ^a^	32.6 ± 1.2 ^a^
*P. ostreatus* 5175 Florida	49.0 ± 0.3 ^d^	5.6 ± 0.8 ^ab^	43.3 ± 0.6 ^b^

The a–d within a column means that significance was determined by one-way ANOVA followed by post-hoc Tukey test (*p* < 0.01). The highest values in bold.

**Table 5:** Antioxidant activities of 80% MeOH extracts of *Pleurotus* species sample extracts dissolved in DMSO.

Sample Name	DPPH		ORAC	CAA Value
	IC_50_ (μg/mL)	mg of TE/(g Extract)	mg of TE/(g Extract)	% of NC
*P. flabellatus* 5013	204.5 ± 45.8 ^a^	24.9 ± 5.4 ^a^	63.9 ± 4.0 ^d^	−1.7 ± 11.3 ^a^
*P. pulmonarius* KZ50	570.9 ± 97.4 ^b^	8.5 ± 1.3 ^b^	35.4 ± 2.2 ^a^	5.9 ± 11.8 ^a^
*P. opuntiae* 5012	390.6 ± 142.7 ^a,b^	13.5 ± 4.6 ^a,b^	36.2 ± 2.4 ^a^	6.6 ± 7.5 ^a^
*P. ostreatus* Sylvan Ivory	552.3 ± 119.0 ^a,b^	9.0 ± 1.8 ^a,b^	44.5 ± 4.5 ^c^	n.d.
*P. ostreatus* 5175 Florida	1134.0 ± 65.8 ^c^	4.3 ± 0.3 ^c^	21.7 ± 4.2 ^b^	6.6 ± 10.29 ^a^
Quercetin (PC)	-	-	-	93.1 ± 4.4
80% MeOH (NC)				−1.7 ± 14.2

The DPPH and ORAC results are expressed as means ± SD for four independent experiments measured in triplicate, while the CAA assay results are given as means ± SE for four independent experiments measured in triplicate. Abbreviations: n.d. = not determined; TE = Trolox equivalent. Values representing the highest activity in bold. The a–d within a column means that significance was determined by one-way ANOVA followed by post-hoc Tukey test (*p* < 0.01).

**Table 6:** Anti-inflammatory activity of 80% MeOH and CHL extracts of *Pleurotus* spp. The protective effect was determined by measuring the extract’s ability to inhibit COX-2 and NF-κB/AP-1 activity.

Sample Name	* COX-2 Average Inhibition ± SD (%)		** NF-κB/AP-1 Activity ± SD (% of NC)
	80% MeOH	CHL	80% MeOH
*P. flabellatus* 5013	28.4 ± 9.4	85.0 ± 0.8 ^a^	83.4 ± 18.4 ^a^
*P. pulmonarius* KZ50	not active	55.6 ± 2.7 ^a,b^	76.6 ± 6.7 ^a^
*P. opuntiae* 5012	2.4 ± 3.7	43.6 ± 12.9 ^b^	80.5 ± 18.6 ^a^
*P. ostreatus* Sylvan Ivory	24.0 ± 6.7	52.8 ± 8.7 ^a,b^	n.d.
*P. ostreatus* 5175 Florida	not active	82.2 ± 2.7 ^a^	72.2 ± 7.7 ^a^
20 μM ibuprofen (PC)	79.0 ± 10.9		-
Prednisone (PC)	-		79.2 ± 8.0 ^a^
80% MeOH (NC)			100.0 ± 12.2 ^b^

The results are expressed as means ± SD for three (*) or four (**) independent experiments measured in triplicate. Abbreviations: n.d. = not determined. The a,b within a column indicates significance as determined by one-way ANOVA followed by post-hoc Tukey test (*p* < 0.01).

## Data Availability

The data presented in this study are available in Table 1, Table 2, Table 3, Table 4, Table 5 and Table 6 and Table S1 and Figures S1–S4.

## Supplementary Materials

The following supporting information can be downloaded at: https://www.mdpi.com/article/10.3390/antiox11081569/s1, Figure S1: PCA of analyzed *Pleurotus* genus samples (5012—*P. opuntiae* 5012; 5175—*P. ostreatus* 5175 Florida; FLAB—*P. flabellatus* 5013; IVORY—*P. ostreatus* Sylvan Ivory; PUL—*P. pulmonarius* KZ50); Figure S2: Cluster analysis of analyzed *Pleurotus* genus samples (5012—*P. opuntiae* 5012; 5175—*P. ostreatus* 5175 Florida*;* FLAB—*P. flabellatus* 5013; IVORY—*P. ostreatus* Sylvan Ivory; PUL—*P. pulmonarius* KZ50), Figure S3: Annotated HPLC−HRMS fragment spectrum of ergothioneine; Figure S4: Tentatively annotated GC chromatogram (a) isomers of 2-butyl-2-octanol (b) dodecanol derivatives (c) 2-hexyl-1-octanol derivatives (d) octadecadienic acid derivatives (e) ergosterol, Table S1: Mass spectrometer parameter settings.

## Sample Availability

Samples of the compounds are available from the authors.

## References

[1] Roncero-Ramos I., Delgado-Andrade C. (2017). The Beneficial Role of Edible Mushrooms in Human Health. Curr. Opin. Food Sci..

[2] Guillamón E., García-Lafuente A., Lozano M., D’Arrigo M., Rostagno M.A., Villares A., Martínez J.A. (2010). Edible Mushrooms: Role in the Prevention of Cardiovascular Diseases. Fitoterapia.

[3] Jo W.-S., Hossain M.A., Park S.-C. (2014). Toxicological Profiles of Poisonous, Edible, and Medicinal Mushrooms. Mycobiology.

[4] Wasser S.P. (2011). Current Findings, Future Trends, and Unsolved Problems in Studies of Medicinal Mushrooms. Appl. Microbiol. Biotechnol..

[5] Patel Y., Narian R., Singh V.K. (2012). Medicinal Properties of Pleurotus Species (Oyster Mushroom): A Review. World J. Fungal Plant Biol..

[6] Hobbs C. (2003). Medicinal Mushrooms: An Exploration of Tradition, Healing, and Culture.

[7] Rama Shankar G.S., Lavekar S.D., Sharma B.K. (2012). Traditional healing practice and folk medicines used by Mishing community of North East India. J. Ayurveda Integr. Med..

[8] Catalogue of Life. https://www.catalogueoflife.org/col/browse/tree/id/857ebe7fdb1404352630eb1d4da99cf1.

[9] Deepalakshmi K., Mirunalini S. (2014). Pleurotus Ostreatus: An Oyster Mushroom with Nutritional and Medicinal Properties. J. Biochem. Technol..

[10] Enshasy H., Maftoun P., Johari H.J., Soltani M., Malik R., Othman N. (2015). The Edible Mushroom *Pleurotus* Spp.: I. Biodiversity and Nutritional Values. Int. J. Biotechnol. Wellness Ind..

[11] Jedinak A., Dudhgaonkar S., Wu Q., Simon J., Sliva D. (2011). Anti-Inflammatory Activity of Edible Oyster Mushroom Is Mediated through the Inhibition of NF-ΚB and AP-1 Signaling. Nutr. J..

[12] Golak-Siwulska I., Kałużewicz A., Spiżewski T., Siwulski M., Sobieralski K. (2018). Bioactive Compounds and Medicinal Properties of Oyster Mushrooms ( *Pleurotus* sp.). Folia Hortic..

[13] Klaus A., Wan-Mohtar W.A.A.Q.I., Nikolić B., Cvetković S., Vunduk J. (2021). Pink Oyster Mushroom Pleurotus Flabellatus Mycelium Produced by an Airlift Bioreactor—the Evidence of Potent in Vitro Biological Activities. World J. Microbiol. Biotechnol..

[14] Murugesan A.K., Gunasagaran K.S. (2021). Purification and Characterization of a Synergistic Bioactive Lectin from Pleurotus Flabellatus (PFL-L) with Potent Antibacterial and in-Vitro Radical Scavenging Activity. Anal. Biochem..

[15] Radzi M.P.M.F., Azizah M., Maininah T., Sumaiyah A. (2021). Growth, Yield And Antioxidant Activity of Grey Oyster Mushroom (Pleurotus Pulmonarius) Grown in Sawdust Substrate With The Supplementation Of Alkaline Materials. JAPS J. Anim. Plant Sci..

[16] Oyetayo V.O., Ogidi C.O., Bayode S.O., Enikanselu F.F. (2021). Evaluation of Biological Efficiency, Nutrient Contents and Antioxidant Activity of Pleurotus Pulmonarius Enriched with Zinc and Iron. Indian Phytopathol..

[17] Nguyen T.K., Im K.H., Choi J., Shin P.G., Lee T.S. (2016). Evaluation of Antioxidant, Anti-Cholinesterase, and Anti-Inflammatory Effects of Culinary Mushroom *Pleurotus Pulmonarius*. Mycobiology.

[18] Xu W.W., Li B., Lai E.T.C., Chen L., Huang J.J.H., Cheung A.L.M., Cheung P.C.K. (2014). Water Extract from *Pleurotus Pulmonarius* with Antioxidant Activity Exerts In Vivo Chemoprophylaxis and Chemosensitization for Liver Cancer. Nutr. Cancer.

[19] Pumtes P., Rojsuntornkitti K., Kongbangkerd T., Jittrepotch N. (2016). Effects of Different Extracting Conditions on Antioxidant Activities of *Pleurotus flabellatus*. Int. Food Res. J..

[20] Smiderle F.R., Olsen L.M., Carbonero E.R., Baggio C.H., Freitas C.S., Marcon R., Santos A.R.S., Gorin P.A.J., Iacomini M. (2008). Anti-Inflammatory and Analgesic Properties in a Rodent Model of a (1→3),(1→6)-Linked β-Glucan Isolated from Pleurotus Pulmonarius. Eur. J. Pharmacol..

[21] Adebayo E., Oloke J., Olusola M., Ajani R., Bora T. (2012). Antimicrobial and Anti-Inflammatory Potential of Polysaccharide from Pleurotus Pulmonarius LAU 09. Afr. J. Microbiol. Res..

[22] Pandey A.T., Pandey I., Kerkar P., Singh M.P. (2021). Antimicrobial Activity and Mycochemical Profile of Methanol Extract from Pleurotus Flabellatus. Vegetos.

[23] Damaris Chinwendu O. (2017). Antioxidant and Antimicrobial Activities of Oyster Mushroom. Am. J. Food Sci. Ant Technol..

[24] Díaz-Godínez G., Téllez-Téllez M., Rodríguez A., Obregón-Barbosa V., Acosta-Urdapilleta M.D.L., Villegas E. (2016). Enzymatic, Antioxidant, Antimicrobial, and Insecticidal Activities of Pleurotus Pulmonarius and Pycnoporus Cinnabarinus Grown Separately in an Airlift Reactor. BioResources.

[25] Kumar K. (2015). Role of Edible Mushrooms as Functional Foods—A Review. South Asian J. Food Technol. Environ..

[26] Sarangi I., Ghosh D., Bhutia S.K., Mallick S.K., Maiti T.K. (2006). Anti-Tumor and Immunomodulating Effects of Pleurotus Ostreatus Mycelia-Derived Proteoglycans. Int. Immunopharmacol..

[27] Xia F., Fan J., Zhu M., Tong H. (2011). Antioxidant Effects of a Water-Soluble Proteoglycan Isolated from the Fruiting Bodies of Pleurotus Ostreatus. J. Taiwan Inst. Chem. Eng..

[28] Reis F.S., Martins A., Barros L., Ferreira I.C.F.R. (2012). Antioxidant Properties and Phenolic Profile of the Most Widely Appreciated Cultivated Mushrooms: A Comparative Study between in Vivo and in Vitro Samples. Food Chem. Toxicol..

[29] Chen S.-Y., Ho K.-J., Hsieh Y.-J., Wang L.-T., Mau J.-L. (2012). Contents of Lovastatin, γ-Aminobutyric Acid and Ergothioneine in Mushroom Fruiting Bodies and Mycelia. LWT.

[30] Calabretti A., Mang S.M., Becce A., Castronuovo D., Cardone L., Candido V., Camele I. (2021). Comparison of Bioactive Substances Content between Commercial and Wild-Type Isolates of Pleurotus Eryngii. Sustainability.

[31] Baek J., Roh H.-S., Baek K.-H., Lee S., Lee S., Song S.-S., Kim K.H. (2018). Bioactivity-Based Analysis and Chemical Characterization of Cytotoxic Constituents from Chaga Mushroom (*Inonotus Obliquus*) That Induce Apoptosis in Human Lung Adenocarcinoma Cells. J. Ethnopharmacol..

[32] Kim H.K., Choi Y.H., Verpoorte R. (2010). NMR-Based Metabolomic Analysis of Plants. Nat. Protoc..

[33] Pedneault K., Angers P., Avis T.J., Gosselin A., Tweddell R.J. (2007). Fatty Acid Profiles of Polar and Non-Polar Lipids of Pleurotus Ostreatus and P. Cornucopiae Var. ‘Citrino-Pileatus’ Grown at Different Temperatures. Mycol. Res..

[34] Mascellani A., Natali L., Cavallini A., Mascagni F., Caruso G., Gucci R., Havlik J., Bernardi R. (2021). Moderate Salinity Stress Affects Expression of Main Sugar Metabolism and Transport Genes and Soluble Carbohydrate Content in Ripe Fig Fruits (*Ficus Carica* L. Cv. Dottato). Plants.

[35] Sharma O.P., Bhat T.K. (2009). DPPH Antioxidant Assay Revisited. Food Chem..

[36] Ou B., Hampsch-Woodill M., Prior R.L. (2001). Development and Validation of an Improved Oxygen Radical Absorbance Capacity Assay Using Fluorescein as the Fluorescent Probe. J. Agric. Food Chem..

[37] Held P. Performing Oxygen Radical Absorbance Capacity Assays with Synergy HT: ORAC Antioxidant Tests. Appl. Note.

[38] Hošek J., Bartos M., Chudík S., Dall’Acqua S., Innocenti G., Kartal M., Kokoška L., Kollár P., Kutil Z., Landa P. (2011). Natural Compound Cudraflavone B Shows Promising Anti-Inflammatory Properties in Vitro. J. Nat. Prod..

[39] Wolfe K.L., Liu R.H. (2007). Cellular Antioxidant Activity (CAA) Assay for Assessing Antioxidants, Foods, and Dietary Supplements. J. Agric. Food Chem..

[40] Malanik M., Treml J., Lelaková V., Nykodymová D., Oravec M., Marek J., Šmejkal K. (2020). Anti-Inflammatory and Antioxidant Properties of Chemical Constituents of Broussonetia Papyrifera. Bioorganic Chem..

[41] McCleary B.V., Draga A. (2016). Measurement of β-Glucan in Mushrooms and Mycelial Products. J. Aoac Int..

[42] Alam N., Amin R., Khan A., Ara I., Shim M.J., Lee M.W., Lee T.S. (2008). Nutritional Analysis of Cultivated Mushrooms in Bangladesh—*Pleurotus Ostreatus*, *Pleurotus Sajor-Caju*, *Pleurotus Florida* and *Calocybe Indica*. Mycobiology.

[43] Chaiharn M., Phutdhawong W.S., Amornlerdpison D., Phutdhawong W. (2018). Antibacterial, Antioxidant Properties and Bioactive Compounds of Thai Cultivated Mushroom Extracts against Food-Borne Bacterial Strains. Chiang Mai J. Sci..

[44] Muan C., Chonju H. (1992). Volatile components of oyster mushrooms (*Pleurotus* sp.) cultivated in Korea. Korean J. Mycol. (Korea Repub.).

[45] Lin P., Yan Z.-F., Kook M., Li C.-T., Yi T.-H. (2022). Genetic and Chemical Diversity of Edible Mushroom Pleurotus Species. BioMed Res. Int..

[46] Avni S., Ezove N., Hanani H., Yadid I., Karpovsky M., Hayby H., Gover O., Hadar Y., Schwartz B., Danay O. (2017). Olive Mill Waste Enhances α-Glucan Content in the Edible Mushroom Pleurotus Eryngii. Int. J. Mol. Sci..

[47] Lam Y.S., Okello E.J. (2015). Determination of Lovastatin, β-Glucan, Total Polyphenols, and Antioxidant Activity in Raw and Processed Oyster Culinary-Medicinal Mushroom, Pleurotus Ostreatus (Higher Basidiomycetes). Int. J. Med. Mushrooms.

[48] Treml J., Večeřová P., Herczogová P., Šmejkal K. (2021). Direct and Indirect Antioxidant Effects of Selected Plant Phenolics in Cell-Based Assays. Molecules.

[49] Bakir T., Karadeniz M., Unal S. (2018). Investigation of Antioxidant Activities of *Pleurotus Ostreatus* Stored at Different Temperatures. Food Sci. Nutr..

[50] Halliwell B., Cheah I.K., Tang R.M.Y. (2018). Ergothioneine—A Diet-derived Antioxidant with Therapeutic Potential. FEBS Lett..

[51] Liang C.-H., Ho K.-J., Huang L.-Y., Tsai C.-H., Lin S.-Y., Mau J.-L. (2013). Antioxidant Properties of Fruiting Bodies, Mycelia, and Fermented Products of the Culinary-Medicinal King Oyster Mushroom, Pleurotus Eryngii (Higher Basidiomycetes), with High Ergothioneine Content. Int. J. Med. Mushrooms.

[52] Kalaras M.D., Richie J.P., Calcagnotto A., Beelman R.B. (2017). Mushrooms: A Rich Source of the Antioxidants Ergothioneine and Glutathione. Food Chem..

[53] Permatasari W., Dayanti D., Khaerunnisa I., Winarni S. (2020). Literatur Review The New Super Antioxidant, Ergothioneine In Pleurotus Ostreatus. Int. J. Health Educ. &Amp; Soc. (IJHES).

[54] Dubost N.J., Beelman R.B., Peterson D., Royse D.J. (2006). Identification and Quantification of Ergothioneine in Cultivated Mushrooms by Liquid Chromatography-Mass Spectroscopy. Int. J. Med. Mushrooms.

[55] Tsiapali E., Whaley S., Kalbfleisch J., Ensley H.E., Browder I.W., Williams D.L. (2001). Glucans Exhibit Weak Antioxidant Activity, but Stimulate Macrophage Free Radical Activity. Free. Radic. Biol. Med..

[56] Mizunoe Y., Kobayashi M., Sudo Y., Watanabe S., Yasukawa H., Natori D., Hoshino A., Negishi A., Okita N., Komatsu M. (2018). Trehalose Protects against Oxidative Stress by Regulating the Keap1–Nrf2 and Autophagy Pathways. Redox Biol..

[57] Radbakhsh S., Ganjali S., Moallem S.A., Guest P.C., Sahebkar A. (2021). Antioxidant Effects of Trehalose in an Experimental Model of Type 2 Diabetes. Natural Products and Human Diseases.

[58] Liu J.-H., Chen M.-M., Huang J.-W., Wann H., Ho L.-K., Pan W.H.T., Chen Y.-C., Liu C.-M., Yeh M.-Y., Tsai S.-K. (2010). Therapeutic Effects and Mechanisms of Action of Mannitol During H_2_O_2_-Induced Oxidative Stress in Human Retinal Pigment Epithelium Cells. J. Ocul. Pharmacol. Ther..

[59] Pelle E., Mammone T., Marenus K., Maes D., Huang X., Frenkel K. (2003). Ultraviolet-B-Induced Oxidative DNA Base Damage in Primary Normal Human Epidermal Keratinocytes and Inhibition by a Hydroxyl Radical Scavenger. J. Investig. Dermatol..

[60] Meza-Menchaca T., Poblete-Naredo I., Albores-Medina A., Pedraza-Chaverri J., Quiroz-Figueroa F.R., Cruz-Gregorio A., Zepeda R.C., Melgar-Lalanne G., Lagunes I., Trigos Á. (2020). Ergosterol Peroxide Isolated from Oyster Medicinal Mushroom, Pleurotus Ostreatus (Agaricomycetes), Potentially Induces Radiosensitivity in Cervical Cancer. Int. J. Med. Mushrooms.

[61] Sinthupoom N., Prachayasittikul V., Prachayasittikul S., Ruchirawat S., Prachayasittikul V. (2015). Nicotinic Acid and Derivatives as Multifunctional Pharmacophores for Medical Applications. Eur. Food Res. Technol..

[62] Tupe R.S., Tupe S.G., Agte V.V. (2011). Dietary Nicotinic Acid Supplementation Improves Hepatic Zinc Uptake and Offers Hepatoprotection against Oxidative Damage. Br. J. Nutr..

[63] Arauz J., Rivera-Espinoza Y., Shibayama M., Favari L., Flores-Beltrán R.E., Muriel P. (2015). Nicotinic Acid Prevents Experimental Liver Fibrosis by Attenuating the Prooxidant Process. Int. Immunopharmacol..

[64] Liu C., Zhao S., Zhu C., Gao Q., Bai J., Si J., Chen Y. (2020). Ergosterol Ameliorates Renal Inflammatory Responses in Mice Model of Diabetic Nephropathy. Biomed. Pharmacother..

[65] Xiong M., Huang Y., Liu Y., Huang M., Song G., Ming Q., Ma X., Yang J., Deng S., Wen Y. (2018). Antidiabetic Activity of Ergosterol from *Pleurotus Ostreatus* in KK-A^y^ Mice with Spontaneous Type 2 Diabetes Mellitus. Mol. Nutr. Food Res..

[66] Taofiq O., Silva A.R., Costa C., Ferreira I., Nunes J., Prieto M.A., Simal-Gandara J., Barros L., Ferreira I.C.F.R. (2020). Optimization of Ergosterol Extraction from *Pleurotus* Mushrooms Using Response Surface Methodology. Food Funct..

[67] Bekiaris G., Tagkouli D., Koutrotsios G., Kalogeropoulos N., Zervakis G.I. (2020). Pleurotus Mushrooms Content in Glucans and Ergosterol Assessed by ATR-FTIR Spectroscopy and Multivariate Analysis. Foods.

[68] Alexandre T.R., Lima M.L., Galuppo M.K., Mesquita J.T., do Nascimento M.A., dos Santos A.L., Sartorelli P., Pimenta D.C., Tempone A.G. (2017). Ergosterol Isolated from the Basidiomycete Pleurotus Salmoneostramineus Affects Trypanosoma Cruzi Plasma Membrane and Mitochondria. J. Venom. Anim. Toxins Incl. Trop. Dis..

[69] Agrawal D., Yin K. (2014). Vitamin D and Inflammatory Diseases. J. Inflamm. Res..

[70] Du B., Lin C., Bian Z., Xu B. (2015). An Insight into Anti-Inflammatory Effects of Fungal Beta-Glucans. Trends Food Sci. Technol..

[71] Deo G.S., Khatra J., Buttar S., Li W.M., Tackaberry L.E., Massicotte H.B., Egger K.N., Reimer K., Lee C.H. (2019). Antiproliferative, Immunostimulatory, and Anti-Inflammatory Activities of Extracts Derived from Mushrooms Collected in Haida Gwaii, British Columbia (Canada). Int. J. Med. Mushrooms.

[72] Echigo R., Shimohata N., Karatsu K., Yano F., Kayasuga-Kariya Y., Fujisawa A., Ohto T., Kita Y., Nakamura M., Suzuki S. (2012). Trehalose Treatment Suppresses Inflammation, Oxidative Stress, and Vasospasm Induced by Experimental Subarachnoid Hemorrhage. J. Transl. Med..

[73] Collins J., Robinson C., Danhof H., Knetsch C.W., van Leeuwen H.C., Lawley T.D., Auchtung J.M., Britton R.A. (2018). Dietary Trehalose Enhances Virulence of Epidemic Clostridium Difficile. Nature.

[74] Jayasuriya W.J.A.B.N., Handunnetti S.M., Wanigatunge C.A., Fernando G.H., Abeytunga D.T.U., Suresh T.S. (2020). Anti-Inflammatory Activity of Pleurotus Ostreatus, a Culinary Medicinal Mushroom, in Wistar Rats. Evid.-Based Complement. Altern. Med..

