# Supplementary material for: Antioxidant and Anti-Inflammatory Activity of Five Medicinal Mushrooms of the Genus Pleurotus
Source: Antioxidants (Basel). 2022 Aug 13;11(8):1569. doi: 10.3390/antiox11081569 (PMC9405179; doi:10.3390/antiox11081569)
Supplement: Supplementary file 1 [file antioxidants-11-01569-s001.zip › antioxidants-1809805-supplementary.pdf]

antioxidants

MDPI

Supplements

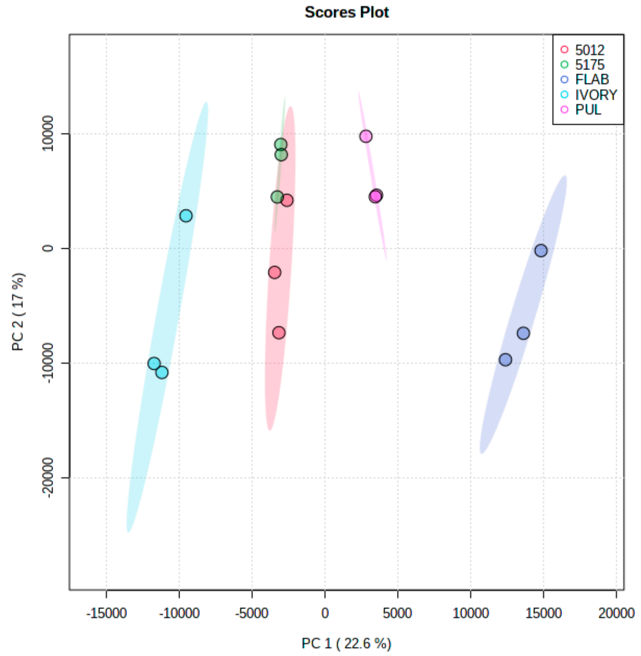

Figure S1. PCA of analyzed genus *Pleurotus* samples (5012 - *P. opuntiae* 5012; 5175 - *P. ostreatus* 5175 Florida; FLAB - *P. flabellatus* 5013; IVORY - *P. ostreatus* Sylvan Ivory; PUL – *P. pulmonarius* KZ50)

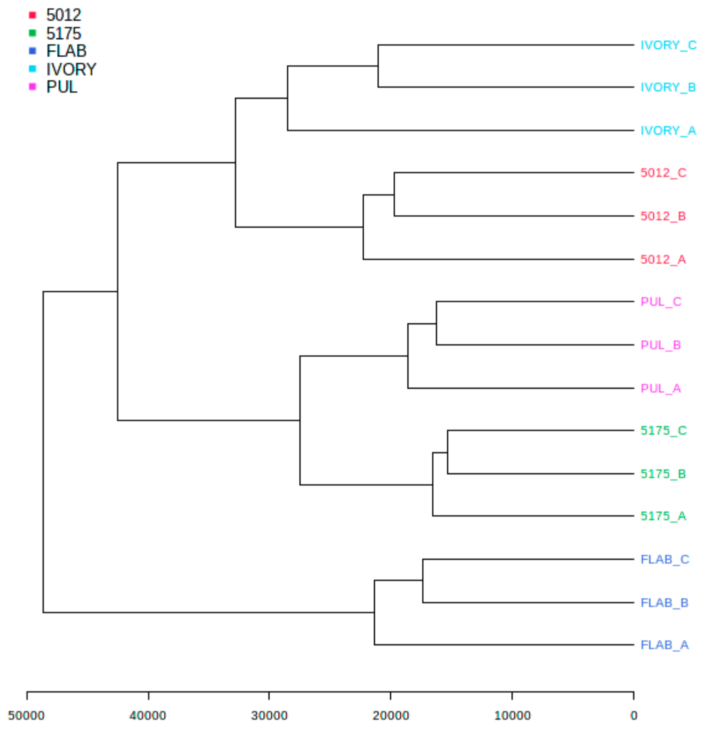

Figure S2. Cluster analysis of analyzed genus *Pleurotus* samples (5012 - *P. opuntiae* 5012; 5175 - *P. ostreatus* 5175 Florida; FLAB - *P. flabellatus* 5013; IVORY - *P. ostreatus* Sylvan Ivory; PUL – *P. pulmonarius* KZ50)

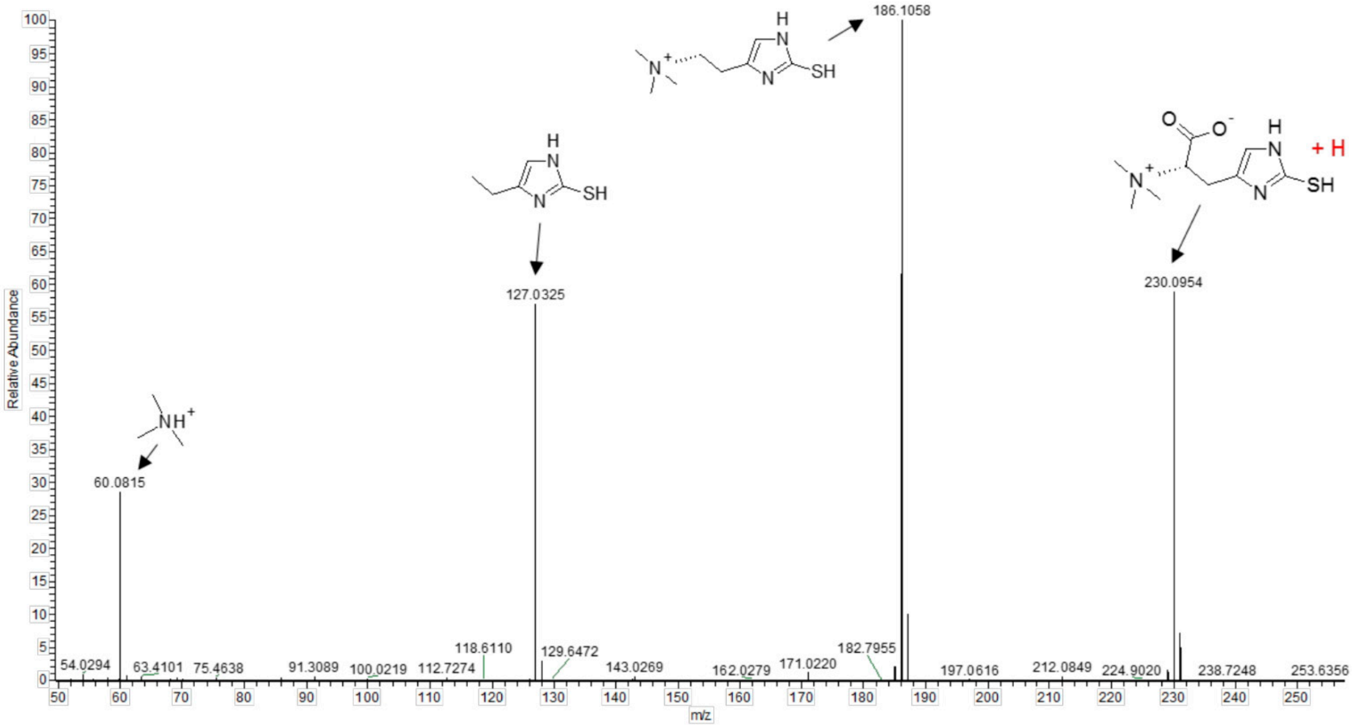

Figure S3. Annotaded HPLC-HRMS fragment spectrum of ergothioneine

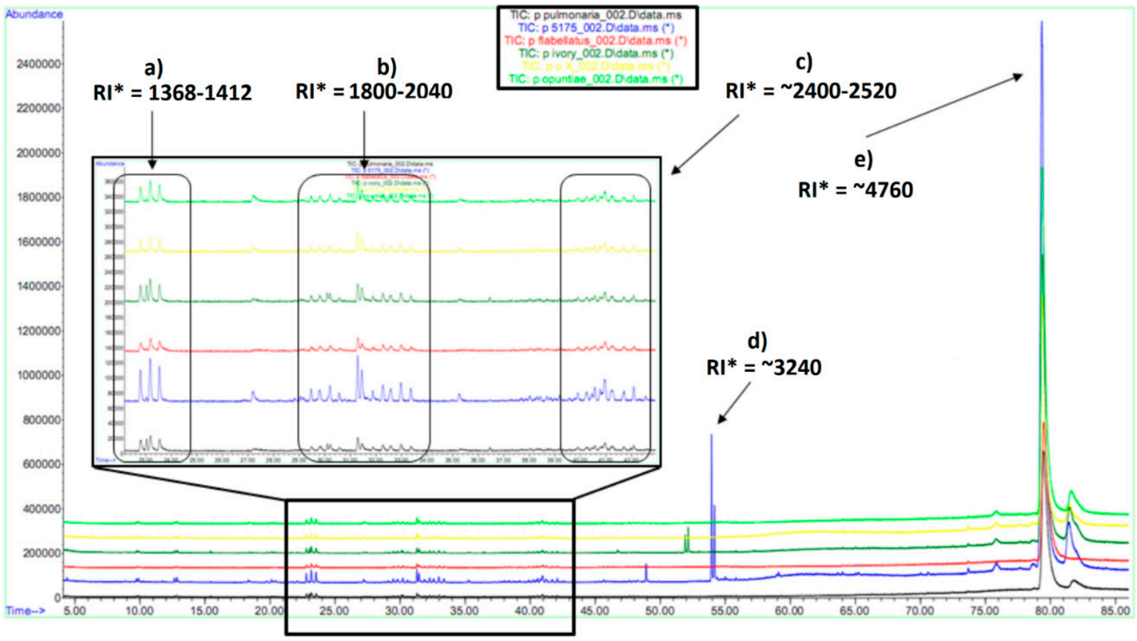

Figure S4. Tentatively annotated GC chromatogram a) isomers of 2-butyl-2-octanol b) dodecanol derivatives c) 2-hexyl-1-octanol derivatives d) octadecadienic acid derivatives e) ergosterol

Table S1. Mass spectrometer parameters settings.

Ion source settings			Acquisition settings		
	ESI	APCI		ESI	APCI
End plate offset (V)	500	500	Mass range (*m/z*)	60 – 1,500	60 – 1,500
Capillary voltage (V)	2,500	4,000	Scan rate (Hz)	1	1
Corona (nA)	-	4,000	Resolution	> 60,000	> 60,000
Nebulizer gas (Bar)	2.0	2.5			
Dry gas (L/min)	5.0	4.0			
Dry temperature (°C)	300	250			
Vaporizer temperature (°C)	-	450			
